# The Contribution of Ultrasonographic Characteristics of Mediastinal Lymph Nodes on Differential Diagnosis of Tuberculous Lymphadenitis from Sarcoidosis

**Published:** 2018-10

**Authors:** Serhat Erol, Ceyda Anar, Onur Fevzi Erer, Can Biçmen, Zekiye Aydoğdu

**Affiliations:** 1 Department of Pulmonary Diseases, Ankara University School of Medicine, Ankara, Turkey,; 2 Department of Chest Diseases, Dr. Suat Seren Chest Diseases and Thoracic Surgery Teaching and Research Hospital, Izmir, Turkey,; 3 Department of Microbiology, Dr. Suat Seren Chest Diseases and Thoracic Surgery Teaching and Research Hospital, Izmir, Turkey,; 4 Department of Pathology, Dr. Suat Seren Chest Diseases and Thoracic Surgery Teaching and Research Hospital, Izmir, Turkey

**Keywords:** Sarcoidosis, Mediastinal tuberculous lymphadenitis, Endobronchial ultrasonography

## Abstract

**Background:**

Sarcoidosis and Mediastinal Tuberculous Lymphadenitis (MTLA) are two granulomatous diseases. Differentiation between these two diseases is dependent on clinical presentation, microbiological investigation, and cytopathological examination. In endemic regions, differential diagnosis of MTLA and sarcoidosis might be difficult. Endobronchial ultrasound guided Transbronchial Needle Aspiration (EBUS-TBNA) is a new diagnostic procedure for the diagnosis of mediastinal lymphadenopathy. EBUS not only enables the sampling of Lymph Nodes (LN), but also visualization of sonographic features of them. We hypothesized that the sonographic features of LN may help to differentiate MTLA from sarcoidosis.

**Materials and Methods:**

This is a retrospective analysis of patients with intrathoracic lymphadenopathy who underwent EBUS-TBNA and were finally diagnosed as sarcoidosis or MTLA. Size, shape, margin, echogenicity, and coagulation necrosis were compared between the groups.

**Results:**

A total of 257 LNs (215 sarcoidosis, 42 MTLA) were examined in 101 patients. A heterogeneous echotexture of lymph nodes was significantly more common (P <0.0001) in MTLA (69%) than sarcoidosis (36.2%). Also, necrosis was statistically significantly higher in MTLA compared to sarcoidosis (P<0.0001). The vascular pattern was similar in both groups (P=0.9050). Nearly half of the patients had grade 1 vascular pattern in both groups. The odds for diagnosis of MTLA were significantly higher in the presence of heterogeneous echotexture (odds ratio [OR], 7,00) or necrosis sign (OR, 131,2).

**Conclusion:**

Vascular patterns of two diseases were similar. Heterogeneous echotexture and necrosis sign in the LNs on EBUS are specific for MTLA. Combination of these findings with a positive tuberculin skin test, favors the diagnosis of MTLA over sarcoidosis.

## INTRODUCTION

Sarcoidosis and Mediastinal Tuberculous Lymphadenitis (MTLA) are two granulomatous diseases. Differentiation between these diseases is dependent on clinical presentation, microbiological investigation for *Mycobacterium tuberculosis* (*M. tuberculosis*), Tuberculin Skin Test (TST) and cytopathological features of granulomas (1, 2).

Conventional Transbronchial Needle Aspiration (TBNA) has long been the available diagnostic procedure (3–5). Recently, Endobronchial ultrasound-guided Transbronchial Needle Aspiration (EBUS-TBNA) became the diagnostic procedure of choice for diagnosis of mediastinal lymphadenopathy, including sarcoidosis and MTLA (6–10). The advent of EBUS not only enabled sampling of Lymph Nodes (LN), but also the visualization of their sonographic features.

In endemic regions, differential diagnosis of MTLA and sarcoidosis might be difficult. The microbiological investigation is highly specific. However, its sensitivity is low. In some situations, stains fail to identify the bacilli. In theory, cytopathological features of sarcoid and tuberculous granulomas are different. The presence of necrosis in granulomas is diagnostic for MTLA. However, sarcoid granulomas might occasionally be necrotizing and tuberculous granulomas non-necrotizing (11).

Studies revealed that EBUS sonographic features of LNs such as round shape, distinct margin, heterogeneous echogenicity, the absence of Central Hilar Structure (CHS) or the presence of necrosis may help to differentiate metastatic LNs from non-metastasic LNs (12–17).

In this study, we hypothesized that the sonographic features of EBUS may help to differentiate MTLA from sarcoidosis.

## MATERIALS AND METHODS

### Patient selection

In this retrospective study, we enrolled patients with a final diagnosis of MTLA or sarcoidosis among the patients who underwent EBUS-TBNA between 2011 and 2015. Size, shape, margin, echogenicity, presence or absence of a central hilar structure or coagulation necrosis were compared between the two groups

### Final diagnosis and distinguishing between tuberculosis and sarcoidosis

The diagnosis of sarcoidosis was made on the presence of consistent clinical and radiological presentation; demonstration of granulomatous inflammation on EBUS without detection of *M. tuberculosis* either with Acid-Fast Bacilli staining (AFB) and/or culture and clinical and radiological response after treatment or spontaneous remission.

Patients were diagnosed as MTLA if there was necrotizing granulomatous inflammation or culture and/or AFB of sampled LNs were positive for *M. tuberculosis*; a positive Nucleic Acid Amplification (NAA) test result with high clinical suspicion of histological changes consistent with tuberculosis (granulomatous inflammation with or without necrosis); or supportive histological changes (granulomatous inflammation without necrosis) with high clinical suspicion and a response to anti-tuberculosis treatment.

### The EBUS-TBNA procedure

All procedures were performed by the same bronchoscopist through the oral route under local anesthesia and conscious sedation with an EBUS-guided TBNA bronchoscope (7.5 MHz, BF-UC160F; Olympus Optical Co Tokyo, Japan). Each LN was punctured at least twice, and tissue core specimens were obtained with a 22-gauge needle. The aspirate was then blown on to a glass slide and also cell blocks were obtained. If no tissue core specimen was obtainable from the initial two aspirations, three or more aspirations of the LN were performed until enough tissue core was obtained.

### Pathologic examination

The aspirate was smeared onto glass slides and stained with Hematoxylin and Eosin (HE). Additionally, cell block was prepared (for histological examination). Rapid On-Site cytological Examination (ROSE) was not available.

### Mycobacterial identification

The samples were suspended in 1 *mL* of Middlebrook 7H9 medium and vortexed. Then, the suspensions were digested and decontaminated by using a commercial decontamination kit; Mycoprosafe (Salubris AS). Mycobacterial cultivation was performed by MGIT 960 system (BD Biosciences, Sparks, MD, USA) according to the recommendations of the manufacturer as described elsewhere (18) and in Lowenstein-Jensen slants (Salubris AS). An AFB smear was prepared from each processed specimen. Differentiation of *M. tuberculosis* and nontuberculous mycobacteria was performed by conventional methods (19) and BD immunochromatographic test (BD Biosciences, Sparks, MD, USA).

### Molecular detection

NAA tests were used for detection of *M. tuberculosis* DNA. The test was the BD ProbeTec ET *M. tuberculosis* (DTB) Assay on the basis of BD ProbeTec ET system (BD Biosciences, Sparks, MD, USA), which utilizes homogenous Strand Displacement Amplification (SDA) technology and fluorescent Energy Transfer (ET). Real-Time PCR was used for molecular detection. The NAA test was performed and evaluated according to the recommendations of the manufacturers.

### Ultrasound image characteristics of LNs

EBUS images of LNs were classified according to previous studies (12, 20).

**Figure 1.**
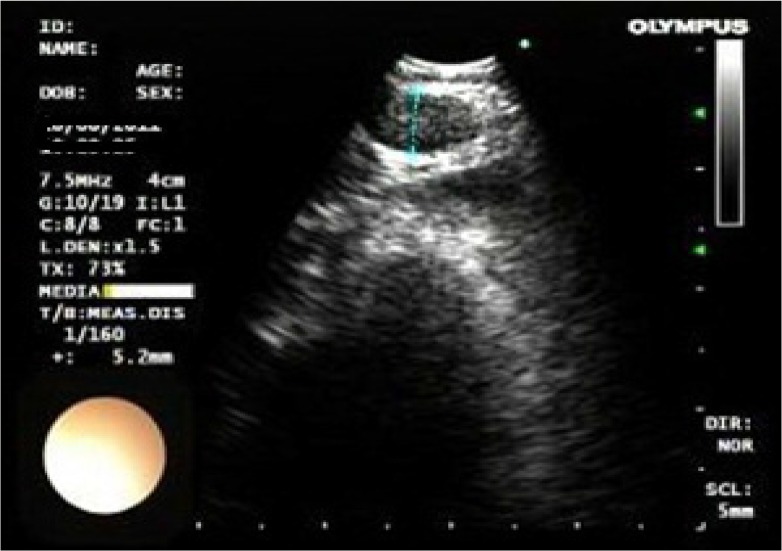
Lymph node with a round shape.

**Figure 2.**
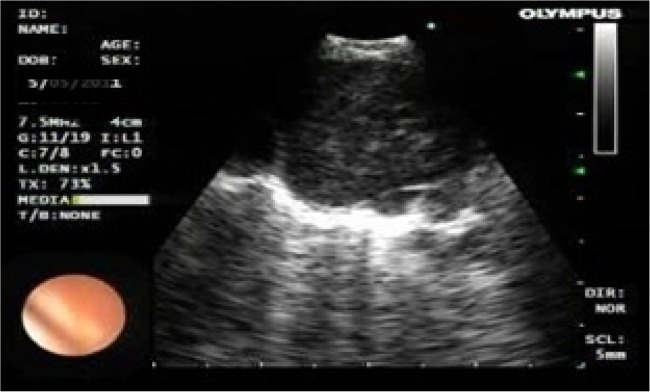
Lymph node with heterogeneous echogenicity.

**Figure 3.**
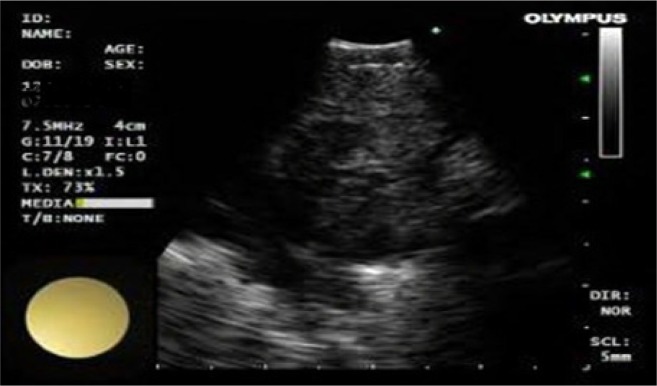
Lymph node with necrosis.

1. Size; short –axis dimension< 1 *cm* or ≥ 1 *cm*,
2. Shape; oval or round (Figure 1)
3. Echogenicity; homogeneous or heterogeneous (Figure 2)
4. Presence or absence of necrosis (Figure 3)

EBUS doppler – mode image characteristics of LNs Vascular pattern characteristics of LNs were categorized based on the study by Nakajima et al. (13):

Grade 0: no blood flow
Grade 1: few vessels
Grade 2: few flow signals and/or few small vessels
Grade 3: rich flow, more than four vessels with different diameters and twist helical flow signal.

### Statistical Analysis

Statistical analysis was performed using the commercial statistical package StatsDirect (version 2.7.2, StatsDirect Ltd, Altrincham, United Kingdom, 2005. http://www.statsdirect.com). Data were expressed as the mean±standard deviation (SD) or number with a percentage. Differences between continuous variables in the 2 groups were evaluated with Mann-Whitney U test; differences between categorical data were compared using the x^2^ test or the Fisher exact test. Multivariate logistic regression analysis was performed to define factors predicting the diagnosis of tuberculosis.

## RESULTS

There were 101 patients. Among them 83 were sarcoidosis and 18 were MTLA. A total of 257 LNs (215 sarcoidosis, 42 MTLA) were examined. Subcarinal LNs were the most sampled station. Patients with sarcoidosis were significantly younger compared to MTLA. The short axis of the LNs on EBUS ranged from 4 to 34 *mm*. The mean short axis of LNs for sarcoidosis and MTLA were 17.3±4.9 and 15.4±5.3, respectively. There wasn’t a statistically significant difference between the two groups regarding the mean short axis of LNs (Table 1).

**Table 1.** Baseline characteristics of patients and the number of sampled lymph nodes

	**Sarcoidosis (n = 83)**	**MTLA (n = 18)**	**Total (n = 101)**	**P value**
Age (years ± SD)	44.6 ±13.4	53.5 ± 16.5	46 ± 14	0.002
Female gender, n(%)	44 (53%)	7 (38.9%)	51 (50.5%)	0.680
Short axis diameter on EBUS,*mm*± SD	17.3 ± 4.9	15.4 ± 5.3	16.3 ± 5.2	0.060
Positive tuberculin skin test	8 (9.6%)	14 (77.7%)	101	0.001
**Sampled lymph node stations**				
Subcarinal	76 (35.3%)	16 (38.1%)	92	
Right lower paratracheal	59 (27.4%)	16 (38.1%)	75	
Left hilar	47 (21.9%)	3 (7.1%)	50	
Left lower paratracheal	7 (3.3%)	5 (11.9%)	12	
Right hilar	26 (12.1%)	2 (4.8%)	28	

SD: standard deviation; EBUS: endobronchial ultrasonography; MTLA: mediastinal tuberculous lymphadenitis.

The percentage of conglomerate LNs was significantly higher in the sarcoidosis group. In both groups, most of the LNs had distinct margin. The percentage of heterogeneous echotexture was 69% in the MTLA group and 36.2% in the sarcoidosis group. The difference was statistically significant (P<0.0001). Also, necrosis was statistically significantly higher in the MTLA group (42.9%) compared to sarcoidosis (2.3%) (P<0.0001). The vascular pattern was similar in both groups (P=0.9050). Nearly half of the patients had grade 1 vascular pattern in both groups (Table 2). Presence or absence of central hilar structure was not available for all patients. Therefore, central hilar structure was excluded from analysis.

**Table 2.** Comparison of sonographic characteristics of lymph nodes in sarcoidosis and mediastinal tuberculous lymphadenitis groups

	**Sarcoidosis n (%)**	**Mediastinal tuberculous lymphadenitis n (%)**	**Odds ratio (95% confidence interval)**	**P value**
Number of sampled lymph nodes	215	42		
**Shape**				
Conglomerate	96 (44.6%)	12 (28.6%)	3.5 (1.4–8.7)	0.0400
Round	72 (33.5%)	14 (33.3%)		
Oval	47 (21.9%)	16 (38.1%)		
**Margins**				
Distinct	173 (80.8%)	28 (66.7%)	1.56 (0.5–5.6)	0.0600
Indistinct	41(19.2%)	14 (33.3%)		
**Echogenity**				
Homogenous	137 (63.8%)	13 (31%)	7.86 (1.98–24.7)	0.0001
Heterogeneous	78 (36.2%)	29 (69%)		
**Necrosis sign**				
Absent	210 (97.7%)	24 (57.1%)	131.2 (18.5–924.7)	0.0001
Present	5 (2.3%)	18 (42.9%)		
**Vascular pattern**				
0	15 (17.7%)	4 (9.5%)	0.9 (0.2–3.1)	0.9050
1	85 (43.6%)	19 (45.3%)		
2	69 (35.4%)	15 (35.7%)		
3	26 (13.3%)	4 (9.5%)		

The odds for diagnosis of MTLA were significantly higher in the presence of heterogeneous echotexture (odds ratio [OR], 7.00) or necrosis sign (OR 131.2) (Table 2). In the MTLA group, diagnostic specificity of heterogeneous echotexture and necrosis signs were 85.5 and 82.7%, respectively. 9.6% of sarcoidosis patients and 77.7% of MTLA patients had TST≥10 *mm*. This difference was statistically significant (P=0.001).

On multivariate analysis, the presence of heterogeneous echotexture and the necrosis signs were significant predictors of the diagnosis of MTLA. A combination of a positive TST with either heterogeneous echotexture or necrosis sign had a specificity of 91% for a diagnosis of MTLA (Table 3).

**Table 3.** Sonographic characteristics of lymph nodes for the differential diagnosis of mediastinal tuberculous lymphadenitis from sarcoidosis

	**Sensitivity % (95% CI)**	**Specifity%(95% CI)**	**PPV% (95% CI)**	**NPV% (95% CI)**
**Heterogeneous echotexture**	60.2(46.4–76.3)	85.5(72.2–90.7)	69(48.3–76.9)	50.2(38.8–70.2)
**Necrosis sign**	42(22.8–55.3)	82.7(76.4–88.2)	67.6(45.8–82.8)	79(71.3–85.8)
**Both heterogeneous echotexture and necrosis sign and TST positivity**	50 (24.5– 60)	91.8 (89.6–96.8)	93.2 (74–97)	83 (75– 90)

CI: confidence interval, NPV: negative predictive value, PPV: positive predictive value; TST: tuberculin skin test

## DISCUSSION

In this study, we compared the EBUS sonographic characteristics of LNs in patients with sarcoidosis and MTLA. The results of this study suggest that heterogeneous echotexture and necrosis in the LNs on EBUS favor a diagnosis of MTLA over sarcoidosis.

A few studies described the ultrasonographic features of LNs in sarcoidosis and MTLA (20–24). Imai et al. (21) reported that low echogenicity and the presence of a germinal center were specific for sarcoidosis. But distinct margin and round shape were similar in both sarcoidosis and malignancy. On the other hand, Ozgul et al. (22) showed that distinct margin and granular appearance were suggestive of sarcoidosis.

Researchers have compared the ultrasonographic features of sarcoidosis and tuberculous lymphadenitis. Dhooria et al. reported that heterogeneous echotexture or necrosis was specific for tuberculosis (20). Rana et al. (23) revealed that patchy hypoechoic or anechoic areas and hyperechoic foci were distinguishing features of tuberculous lymph nodes compared to sarcoidosis. Fritscher-Ravens et al. stated that inhomogenous, hyperechoic areas were more common in tuberculosis compared to sarcoidosis. Vascularity was increased in sarcoidosis compared to MTLA (24).

In our study, there wasn’t any statistically significant difference between sarcoidosis and MTLA regarding size and margins of LNs. As previously stated (20), a distinct margin might be a common feature of all lymphadenopathies irrespective of the underlying disease. Conglomerate LNs were considered in favor of sarcoidosis, while oval shape was higher in the MTLA group in our study. Consistent with previous studies, we found that homogeneous echogenicity was statistically significantly higher in sarcoidosis. While the presence of heterogeneous echotexture had a specificity of 85.5% for the diagnosis of MTLA. Also, necrosis sign was an important sonographic feature that may differentiate MTLA from sarcoidosis. Necrosis sign had a high specificity but low sensitivity for the diagnosis of MTLA. But necrosis may also be seen in malignant involvement (15, 23). Therefore in endemic areas, differential diagnosis of MTLA from malignancy may be difficult.

Similar to previous studies, we accepted TST≥10 *mm* as positive in order to provide a comparison point for our results with previous studies. The percentage of patients with a TST≥10 *mm* was significantly higher in the MTLA group. However, TST cut-off differs according to the underlying disease. For example, in patients with underlying immunosuppression, TST is positive if ≥5 *mm* or TST≥10 *mm* in patients with the end-stage renal disease. Furthermore, TST can be false positive in patients who were vaccinated with Bacille Calmette–Guerin (BCG) (25).

Previous studies (13, 26) have demonstrated that vascular image patterns of LNs may help to differentiate malign LNs from those that are benign. However, we did not find any statistically significant difference between the vascular patterns of MTLA and sarcoidosis. We think that increased vascularity and rich flow is a consequence of increased angiogenesis in metastatic LNs. Therefore different vascular patterns between benign and malignant LNs are not surprising. However, MTLA and sarcoidosis are both granulomatous diseases with similar inflammatory processes and therefore vascular patterns were similar in our study.

Our study has some limitations. First, it’s a retrospective, single-center study with a limited number of patients, especially in the MTLA group. In addition, we did not follow any numeric criterion for defining the shape.

## CONCLUSION

In conclusion, the sonographic features of LNs may be reassuring for the final diagnosis. Heterogeneous echotexture and necrosis in LNs on EBUS are specific for MTLA.
